# A database of metazoan cytochrome c oxidase subunit I gene sequences derived from GenBank with CO-ARBitrator

**DOI:** 10.1038/sdata.2018.156

**Published:** 2018-08-07

**Authors:** Philip Heller, James Casaletto, Gregory Ruiz, Jonathan Geller

**Affiliations:** 1 Moss Landing Marine Laboratories, Moss Landing, California 94039, USA; 2 San Jose State University, San Jose, California 95192, USA; 3 Smithsonian Environmental Research Center, Edgewater, Maryland 21037, USA

**Keywords:** Classification and taxonomy, Data mining, Mitochondrial genome, Biodiversity

## Abstract

The Cytochrome C Oxidase subunit I gene (“COI”) is the *de facto* standard for animal DNA barcoding. Organism identification based on COI requires an accurate and extensive annotated database of COI sequences. Such a database can also be of value in reconstructing evolutionary history and in diversity studies. Two COI databases are currently available: BOLD and Midori. BOLD’s submissions conform to stringent sequence and metadata requirements; BOLD is specific to COI but makes no attempt to be comprehensive. Midori, derived from GenBank, has more sequences but less stringent standards than BOLD, resulting in higher error rates. To address the need for a comprehensive and accurate COI database, we adapted the ARBitrator algorithm, which classifies based only on sequence properties and has successfully auto-curated bacterial genes mined from GenBank. The adapted algorithm, which we call CO-ARBitrator, built a database of over a million metazoan COI sequences. Sensitivity and specificity are significantly higher than Midori. Specificity is comparable to what BOLD achieves with data quality prerequisites. Results and software are publicly available.

## Background & Summary

Traditional taxonomic identification based on observable traits is rapidly being replaced by molecular approaches. Sequence analysis has two advantages over observation of morphology. No human taxonomist is required, so human error is eliminated; moreover, genetically distinct cryptic species, which cannot be visually distinguished^1,2^ are easily recognized.

The success of molecular approaches prompted the concept of a “barcode of animal life” – a gene whose sequence reliably and uniquely identifies most animals. In 2002, Hebert proposed cytochrome *c* oxidase I (COI) as a standard for molecular barcoding of animals^3,4^. COI is well suited for this purpose. Reliable primers are available^5–7^; COI is present in all animals; it has no introns; indel mutations are rare; and it has a high substitution rate in the 3^rd^ codon position, providing nucleotide sequence diversity.

The Consortium for the Barcode of Life (CBOL) was founded in 2004, with the mission to build a barcode sequence library for all eukaryotic life. In 2007, CBOL released the Barcode Of Life Data System (BOLD), including a growing database of curated COI sequences^8^. All records are isolated using trusted PCR primers, are supported by voucher tissue samples^9^ and collection records, have length ≥ 500 bp, and are accepted by a COI Hidden Markov Model. BOLD is expected to grow to 100 million records, representing 10x coverage for 10 million animal species^8^. Sequences are periodically deposited with GenBank.

BOLD’s stringent curation protocol promotes specificity but limits growth. GenBank^10^ contains large numbers of COI sequences that are not part of BOLD. Informatic species identification could benefit from access to these sequences, but their retrieval from GenBank is difficult. The Midori-UNIQUE database^11^ contains approximately 580,000 COI nucleotide records selected from GenBank primarily on the basis of annotation. However, annotations can be unreliable, due to human error and propagation of errors by annotation software^12^. Sequence-based classification is more reliable but isn’t straightforward: simple BLAST searches^13^ aren’t effective, because no alignment score or E-value cutoff is diagnostic across all animal clades.

Our previous success with using the ARBitrator algorithm to mine GenBank for the prokaryotic *nifH* gene^14^ suggests that ARBitrator might be adapted to curating COI. The algorithm begins with a sensitivity phase in which representative sequences of the target gene are submitted as BLASTP queries against GenBank with a loose E-value threshold. Records found are candidates for acceptance; they are passed to a specificity phase and retained if a reverse-PSI BLAST search^15^ of GenBank’s Conserved Domain Database^16,17^ establishes similarity to a conserved domain of the target gene and relatively low similarity to any other domain. When targeted to *nifH*, the algorithm identified tens of thousands of records with very low error rates; indeed error rates were orders of magnitude less than those of a Hidden Markov Model pipeline^18^.

Adaptation to a eukaryotic gene such as COI had not been accomplished before the work presented here and would likely present scaling challenges, since the number of COI records in GenBank is an order of magnitude greater than the number of *nifH* records. The algorithm requires user specification of known sequences of the gene of interest that broadly represent the gene’s diversity; in the case of COI, this means selection of representatives across all metazoa. The algorithm also requires a negative training set for parameter tuning; paralogs of the gene of interest are effective negative training set members, but COI has no known paralogs other than the *numt* pseudogenes^19,20^. Lastly, ARBitrator requires classifying relevant conserved domains as either diagnostic of the gene of interest, anti-diagnostic, or uninformative; previously this has involved fewer than 10 conserved domains, but over 700 COI conserved domains have been defined. If these challenges could be overcome, sequence-based mining of GenBank could produce a COI collection that is more sensitive than BOLD but comparably specific, while also being both more sensitive and more specific than Midori. Such a database would improve species identification, and could also inform studies of diversity and evolutionary history.

Here we report successful adaptation of ARBitrator to the curation of animal COI records. We call the adapted algorithm “CO-ARBitrator”. CO-ARBitrator has identified 1,054,973 records in GenBank, of which 507,651 are not in the BOLD database, 491,465 are not in the Midori database, and 123,412 are not in BOLD or Midori.

## Methods

The ARBitrator heuristic begins with a “sensitivity phase”, in which candidate amino acid sequences are retrieved from GenBank; then a “specificity phase” classifies candidates. In the sensitivity phase, representative amino acid sequences, chosen to broadly represent the diversity of the gene of interest, are submitted as BLASTP queries against GenBank. Subjects are retained into the pool of candidates if their quality exceeds a predetermined threshold, where quality is defined as the negative log-10 of the smallest E-value among all hits to the subject. (Example: if 2 representatives hit a subject with E-values of 1.0e-50 and 1.0 e-40, then the subject’s quality is 50.) In the specificity phase, candidates are submitted as RPSBLAST queries against GenBank’s Conserved Domain Database. Subjects are classified as being the gene of interest if their superiority exceeds a predetermined threshold. Superiority is defined as the negative log 10 of the E-value of the best match to a conserved domain associated with the gene of interest, minus the negative log 10 of the E-value of the best match to a conserved domain associated with a different gene. (Example: if a candidate hits a conserved domain of the gene of interest with E-value of 1.0e-70, and hits a conserved domain of a different gene with E-value of 1.0e-65, then the candidate’s superiority is 5). The quality and superiority thresholds are determined by analysis of hand-selected positive and negative training sets.

To generate a representative set of BLASTP queries that captures the genetic diversity of the COI gene, complete COI nucleotide sequence collections for each of 10 major metazoan phyla (Annelida, Arthropoda, Bryozoa, Chordata, Cnidaria, Echinodermata, Mollusca, Nematoda, Platyhelminthes, and Porifera) were downloaded from the BOLD database. Each phylum was independently explored using simulated annealing software^21,22^ to select approximately 20 sequences from each phylum with approximately maximum mutual p-distance within the phylum. For each representative nucleotide sequence, the most similar amino acid sequence in the GenBank nr database was determined by BLASTX search; if this amino acid sequence contained a COI conserved domain, it was added to the CO-ARBitrator representative set (CO-ARBitrator representative COI protein sequences, Data Citation 1).

To collect a positive training set of COI amino acid sequences, random BOLD COI nucleotide records for each of the major metazoan phyla listed above were submitted as BLASTX queries against the GenBank nr database, and the most similar amino acid record was added to the training set; the process was repeated until the set contained at least 230 members of each phylum, with a total of 3055 members overall (CO-ARBitrator positive training set, Data Citation 1). To collect a negative training set, random members of the positive training set were submitted as BLASTP queries against the GenBank nr database, and best scoring matches not annotated as COI were retained. These were then submitted as BLASTP queries against nr to generate a negative training set of 495 records of CcmD, FoxA, Glycosyl Transferase, Laglidadg, and Trimethyllysine dioxygenase. (CO-ARBitrator negative training set, Data Citation 1). Quality and superiority thresholds of 0.9 and 5 respectively, yielding no errors on either training set, were selected by exhaustive search of {quality, superiority} space, to optimize sensitivity and specificity in acceptance of positive training set members and rejection of negative training set members. The thresholds were verified by N-fold cross validation.

Relevant conserved domains were identified by submitting all members of the representative set as RPSBLAST queries to the GenBank conserved domain database. Matching domains were manually classified as positive (i.e. indicative of COI), negative, or uninformative.

**Figure 1:**
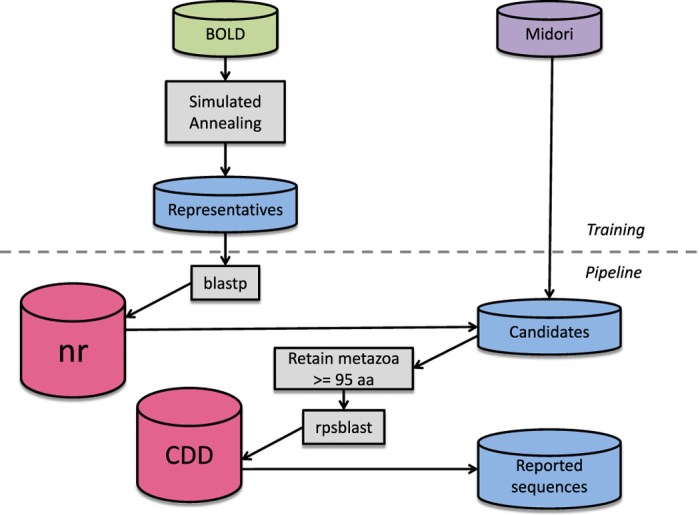
CO-ARBitrator data flow.

The original ARBitrator Java code was modified to support the large representative set and conserved domain list associated with COI, and to reject non-metazoan sequences and sequences with length <95 aa (amino acids). (The algorithm loses accuracy with shorter sequences, perhaps because shorter sequences have low probability of containing diagnostic conserved domains). The modified code base (Fig. 1) has been renamed “CO-ARBitrator”. The GenBank non-redundant protein database (nr) was downloaded on June 7, 2017, and CO-ARBitrator was run with the quality and superiority thresholds determined as described as above. After the quality phase, protein record equivalents of all non-pseudogene nucleotide sequences in the Midori-UNIQUE collection were added to the set of candidates; the superiority phase was then run to report candidates classified as COI. For candidate records not taken from the Midori collection, nucleotide sequences were retrieved by scanning for the FEATURES.CDS./coded_by field, and extracting the coding sequence from the indicated nucleotide record. BOLD sequences were not added to the candidate set because the objective was to mine GenBank for COI sequences, and at any moment not all BOLD records have been deposited in GenBank.

CO-ARBitrator was then run again with quality and superiority thresholds relaxed to 0 and -20 respectively; sequences accepted only by the relaxed thresholds (but rejected by the actual thresholds, hence classified as non-COI) were used for computation of the algorithm’s false negative rate. False positive and false negative rates, conditioned on annotation accuracy, were computed by software retrieval of sequence annotations followed by manual classification of all annotations. To determine which accepted sequences originated from the BOLD database, the DBSOURCE value of each accepted sequence was retrieved and scanned for the string */db_xref="BOLD*: followed by *COI* in its FEATURES.source field.

CO-ARBitrator classified 1,054,973 metazoan GenBank protein sequences with length ≥ 95 aa as being COI. A total of 855,854 of the sequences had binomial identifications, 471 had only genus-level identifications, and 199,119 had higher-level identifications. Results are published as a protein fasta file, a nucleotide fasta, and a comma-separated-values file containing protein and nucleotide accession numbers.

### Code availability

The CO-ARBitrator source code is available for download, along with instructions for installation and execution (Coarbitrator code release 1.0, Data Citation 1); it is also available from GitHub at https://github.com/PhilipHeller/CO-ARBitrator. The code is provided under the terms of the GNU General Public License as published by the Free Software Foundation.

## Data Records

Amino acid sequences resulting from the June 7, 2017 CO-ARBitrator run are available at (CO-ARBitrator amino acid records, fasta format, Data Citation 1). Deflines consist of a protein accession number, then a binomial identification, then a taxonomy; these fields are delimited by double underscores. The binomial identification and the taxonomy are internally delimited by semicolons. Users who wish to target a particular taxon can perform a text search in the file for the name of the taxon of interest, and retain records that match the search. For example, the following LINUX command would output amino acid records from genus *Watersipora*:

grep –A 1 Watersipora Coarbitrator_COI_aa.faa

Nucleotide sequences resulting from the June 7, 2017 CO-ARBitrator run are available at (CO-ARBitrator COI nucleotide records, fasta format, Data Citation 1). Deflines consist of a nucleotide accession number, then a binomial identification, then a taxonomy; these fields are delimited by double underscores. The binomial identification and the taxonomy are internally delimited by semicolons. Users who wish to target a particular taxon can perform a text search in the file as shown above.

Amino acid and nucleotide accession numbers resulting from the June 7, 2017 CO-ARBitrator run are available at (Coarbitrator COI protein and nucleotide accession numbers, CSV format, Data Citation 1). The first field of each line is an amino acid accession number; the second field is the corresponding nucleotide accession number or “n/a” if the nucleotide accession number is not available. Lines are delimited by commas.

## Technical Validation

CO-ARBitrator accepted 1,054,973 sequences, of which 1,054,305 were annotated as COI, 36 were annotated as not COI, and 632 had ambiguous annotations. Thus the CO-ARBitrator false positive rate, conditioned on annotation accuracy, was 0.0034%. However, of the 36 sequences annotated as not COI, none had measurable similarity to any non-COI conserved domains. If some of these sequences were misannotated and are in fact COI, the false positive rate is less than 0.0034% and may in fact be undetectable.

**Figure 2:**
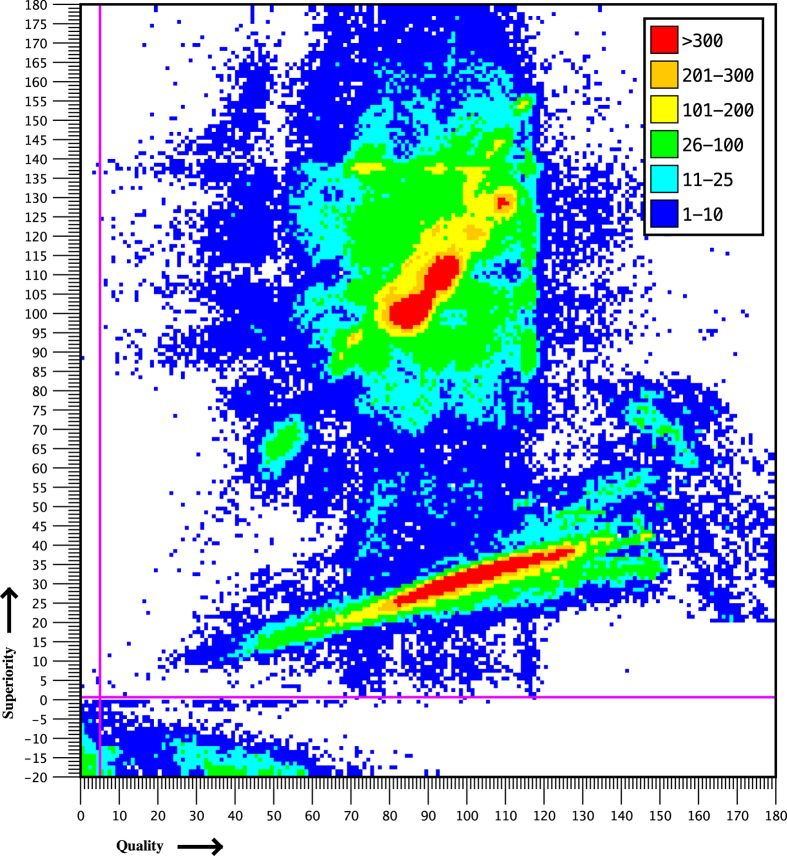
Distribution of accepted/rejected metazoan sequences with length ≥95 aa.

When the quality and superiority thresholds were relaxed, 293,692 additional metazoan sequences were recovered (upper-left, lower-left, and lower-right quadrants of Fig. 2 as defined by the magenta hairlines). Only 154 of these sequences were annotated as COI, 280,048 were annotated as not COI, and 3,493 had ambiguous annotations. Thus the false negative rate, conditioned on annotation accuracy, was 0.054%. However, of the 154 records annotated as COI, only 5 had positive superiority. The remaining 149 had negative superiority that is <−16; the most common best-matched conserved domains were COX2 (86 sequences) and CYTB. If some of these sequences were misannotated and are in fact COI, the false negative rate is less than 0.054% and may be in fact as low as 0.0018%. This low rate validates the diversity of the representative set of initial BLAST query sequences, and supports the validity of the simulated annealing approach to selecting the representatives.

CO-ARBitrator rejected 19,021 of the 582,529 sequences accepted by Midori. Of these, there were 7,191 shorter than the CO-ARBitrator length threshold of 95 aa. Of the remaining 11,830 records, 10,825 records had negative superiority, due to highest conserved domain similarity to a non-COI conserved domain. There were 10,811 records that were most similar to the ND1 or ND2 (NADH dehydrogenase) domains. Thus the Midori false-positive rate conditioned on CO-ARBitrator accuracy was 1.86%.

CO-ARBitrator accepted 491,465 records that Midori rejected. Of these, there were 198,950 that lacked binomial identification and hence were not eligible for inclusion in the Midori collection. Of the remaining 292,515 records, which all had binomial identification, 211 records had sequences that were identical to a Midori sequence for the same species. There were 292,314 CO-ARBitrator sequences with binomial identification that were not identical to any Midori sequence. Of these, there were 41,724 sequences that were deposited with GenBank before Midori’s snapshot on Sept 18, 2015. There were 39,869 unique records that could be considered as Midori false negatives.

CO-ARBitrator accepted 507,651 sequences that were absent from BOLD. CO-ARBitrator rejected 1,762 sequences that were present in BOLD. Of these, there were 1,716 that were shorter than the CO-ARBitrator length threshold (95 aa). The remaining 46 BOLD records that CO-ARBitrator rejected had length above the CO-ARBitrator threshold, had strongly negative superiority (−48.8 through −26.8), were most similar to the COX2 conserved domain, and were annotated as “cytochrome oxidase subunit 2”; 40 of these sequences were submitted to BOLD from a single study, and the remaining 6 sequences were submitted from 2 studies.

Figure 2 shows a heat map of the number of metazoan sequences with length ≥95 aa, for each (quality/superiority) pair, detected by CO-ARBitrator with relaxed thresholds. Magenta hairlines indicate the quality and superiority thresholds. Sequences above and to the right of the hairlines are accepted, while sequences below and/or to the left are rejected but used for calculation of the false negative rate. Linear regression of the points shows a linear relationship with coefficient of determination=0.014. The weakness of correlation between quality and superiority demonstrates the need for classification based on both statistics.

CO-ARBitrator’s sequence-based COI classification is more sensitive and more specific than Midori’s annotation-based classification. It is also more sensitive than the BOLD protocol while offering comparable specificity. We note, however, that BOLD can potentially receive the COI sequence of any living organism, while CO-ARBitrator can only recover sequences that have previously been deposited with GenBank. At any moment, BOLD may contain many records that are not in GenBank and hence are not accessible to CO-ARBitrator. However, these records will be deposited to GenBank during the next periodic update from BOLD, and will be added to the CO-ARBitrator collection during a subsequent run.

The efficacy of the ARBitrator algorithm has now been demonstrated on 2 bacterial genes (*nifH* and *nifD*) and one mitochondrial gene (COI). The algorithm can adapted to other genes by selecting appropriate representative, positive and negative training sets, identifying conserved domains which are positively and negatively diagnostic, and computing optimum quality and superiority thresholds.

## Additional information

**How to cite this article**: Heller, P. *et al*. A database of metazoan cytochrome c oxidase subunit I gene sequences derived from GenBank with CO-ARBitrator. *Sci. Data* 5:180156 doi: 10.1038/sdata.2018.156 (2018).

**Publisher’s note**: Springer Nature remains neutral with regard to jurisdictional claims in published maps and institutional affiliations.

## Supplementary Material
